# Scoring balloon versus plain balloon vessel preparation before drug-coated balloon angioplasty for femoropopliteal atherosclerotic occlusive disease: a two-centre retrospective cohort study

**DOI:** 10.3389/fcvm.2026.1975976

**Published:** 2026-10-14

**Authors:** Lei Qi, Hongzhi Sun, Yaying Wu, Yihao Wang, Jiannan Wang, Yizhan Shi, Zhijian Dai, Jiahe Zhang, Shuangshuang Li, Zan Zeng, Jian Zhou

**Affiliations:** 1 Oriental Pan-Vascular Devices Innovation College, University of Shanghai for Science and Technology, Shanghai, China; 2 Department of Vascular Surgery, The Third Affiliated Hospital of Naval Medical University, Shanghai, China; 3 Department of Interventional Vascular, The Third People’s Hospital of Hefei (Hefei Third Clinical College of Anhui Medical University), Hefei, Anhui, China; 4 Department of Vascular Surgery, Taicang TCM Hospital Affiliated to Nanjing University of Chinese Medicine (Taicang Hospital of Traditional Chinese Medicine), Taicang, China; 5 Department of Vascular Surgery and Intervention, The Fourth Affiliated Hospital of Soochow University, Suzhou, Jiangsu, China

**Keywords:** drug-coated balloon, occlusive femoropopliteal disease, plain balloon, scoring balloon, vessel preparation

## Abstract

**Background:**

Optimal vessel preparation before drug-coated balloon (DCB) angioplasty in femoropopliteal atherosclerotic occlusive disease remains counteracted by dissection and recoil. We evaluated whether scoring balloon (SB) vessel preparation improves procedural immediate effect and mid-term outcomes compared with plain balloon (PB).

**Methods:**

Patients with femoropopliteal occlusive disease undergoing DCB angioplasty following SB or PB preparation (2022-2025) across two tertiary centers were reviewed. 1:1 propensity score matching (PSM) was implemented. Primary endpoint was target vessel dissection. Secondary endpoints included bailout stenting, procedural complications, ankle-brachial index (ABI), 12-month primary patency, and freedom from clinically driven target vessel revascularisation (CD-TVR).

**Results:**

After matching, 63 patients were in each group. Target vessel dissection occurred in 49.2% of Group PB and 17.5% of Group SB (OR: 5.00, 95% CI: 1.91–13.06, *p* = 0.001). For Groups PB and SB, bailout stenting rates were 31.7% and 9.5%, respectively (OR: 3.80, 95% CI: 1.42–10.17; *p* = 0.008). Procedural complication rates were 6.3% and 3.2% (OR: 2.00, 95% CI: 0.37–10.92; *p* = 0.424). 12-month primary patency were 87.3% and 88.9% (HR: 1.18, 95% CI: 0.44–3.22; *p* = 0.739). 12-month freedom from CD-TVR were 88.9% and 93.7% (HR: 1.83, 95% CI: 0.52–6.47; *p* = 0.348).

**Conclusion:**

In this propensity-matched cohort, SB vessel preparation before DCB angioplasty was associated with lower rates of target vessel dissection and bailout stenting than PB. No statistically significant difference in the prespecified procedural complications, 12-month patency or CD-TVR was observed.

## Introduction

The femoropopliteal segment is commonly affected by lower-limb atherosclerotic disease and remains difficult to treat because of the flexion, torsion, and compression to which it is repeatedly exposed. These biomechanical forces contribute to restenosis and may compromise the durability of permanent implants (1–4). Contemporary guidelines support percutaneous transluminal angioplasty (PTA) as a principal endovascular treatment for femoropopliteal disease, with stent implantation generally reserved for bailout indications (5–7).

Drug-coated balloon (DCB) improves patency and reduce restenosis after lower-limb endovascular intervention (8–11). Nevertheless, treatment failure remains a concern in severely calcified and long chronic occlusions, making adequate vessel preparation and the prevention of severe dissection and recoil particularly important (12). Plain balloon (PB) remains the conventional preparation strategy, although its performance may be inconsistent in complex occlusive, calcified, or recoil-prone lesions (13, 14).

Scoring balloon (SB) incorporates external scoring elements that concentrate mechanical force at discrete contact points and may therefore permit more controlled plaque modification with less vessel-wall injury (15). Existing SB studies differ substantially in patient selection, calcification burden, lesion length, device design, and endpoint definition, and comparative real-world data remain limited (16, 17). This study was restricted to complete femoropopliteal occlusions and compared SB plus DCB with PB plus DCB for the immediate vascular response, bailout stent requirement, periprocedural safety, and 12-month outcomes (Figure 1).

**Figure 1:**
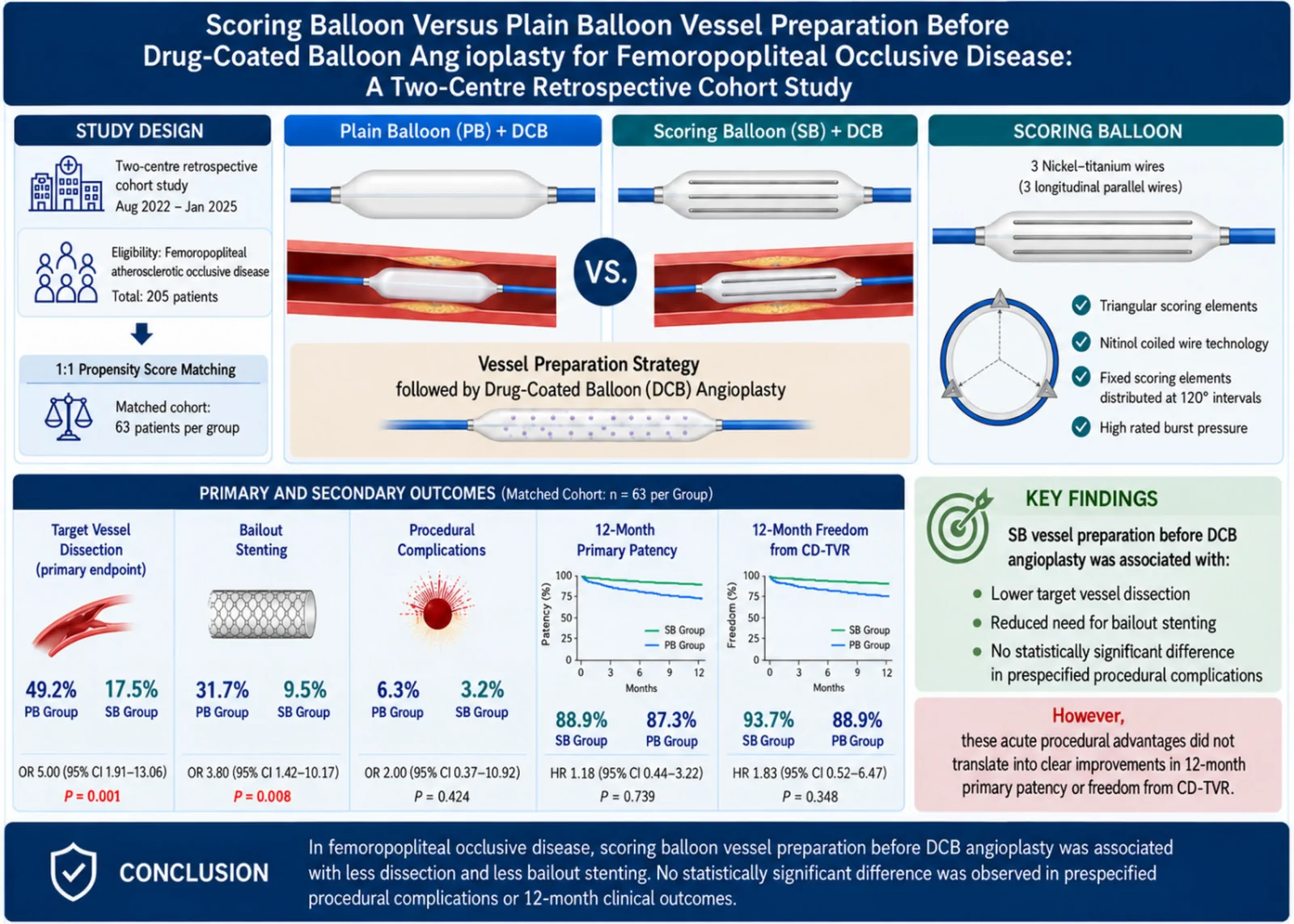
Graphical abstract. CD-TVR, clinically driven target vessel revascularisation; CI, confidence interval; DCB, drug-coated balloon; HR, hazard ratio; OR, odds ratio; PB, plain balloon; SB, scoring balloon.

## Materials and methods

### Patients

This two-centre retrospective cohort study consecutively included patients who underwent PB followed by DCB angioplasty or SB followed by DCB angioplasty for femoropopliteal disease at two large tertiary hospitals in China from August 2022 to January 2025. Patients were classified as Group PB or Group SB according to the vessel preparation strategy used during the index procedure. The inclusion criteria were: (1) Femoropopliteal atherosclerotic complete occlusive disease confirmed by lower-limb computed tomography angiography (CTA); (2) Rutherford categories 3–6; (3) Treatment of a unilateral femoropopliteal lesion; (4) At least one patent distal runoff vessel. The exclusion criteria were: (1) Acute lower-limb arterial embolism; (2) Femoropopliteal in-stent restenosis; (3) Incomplete records. Flowchart of the study (Figure 2).

**Figure 2:**
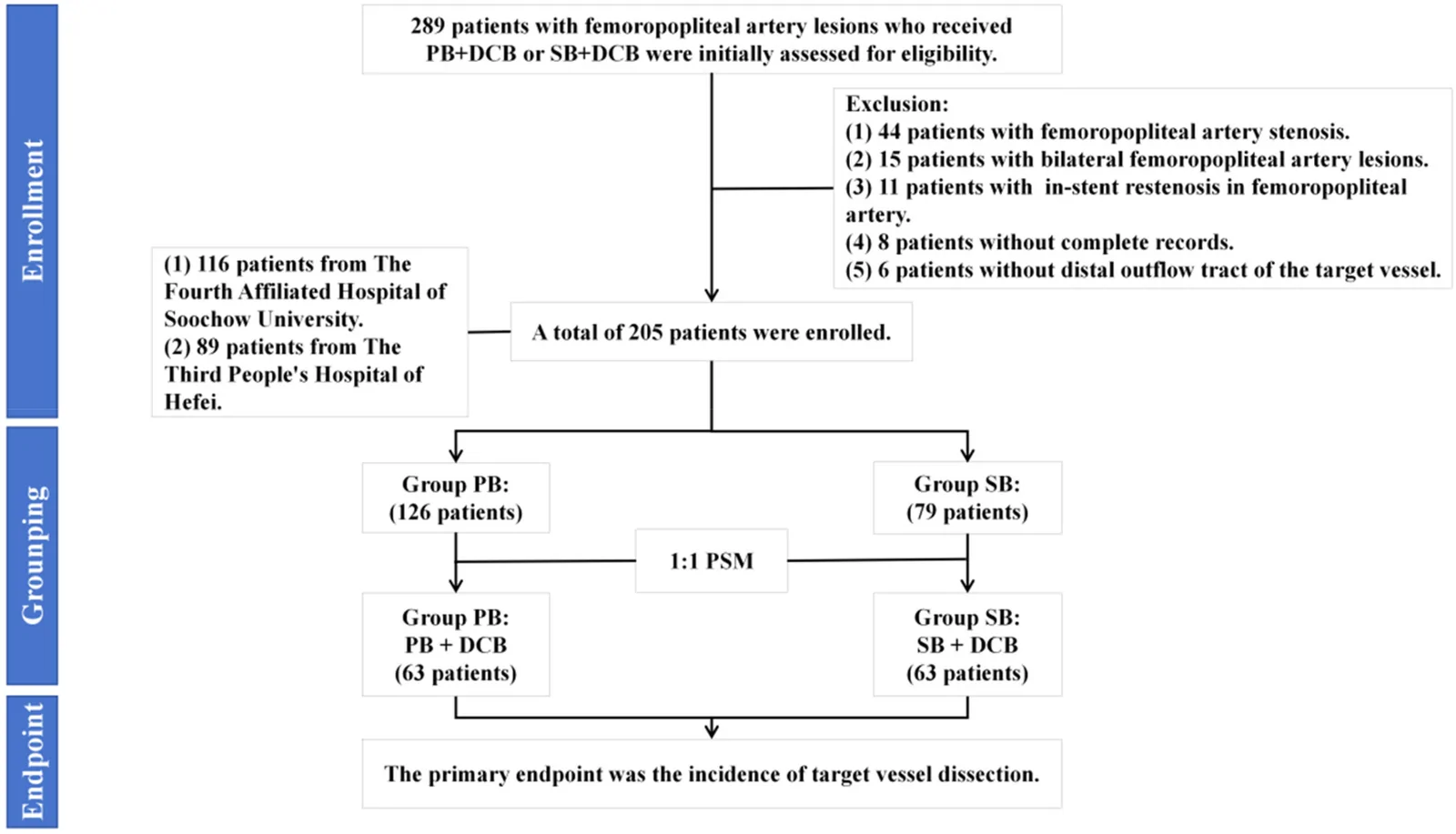
Flowchart of the study. DCB, drug-coated balloon; PB, plain balloon; PSM, propensity score matching; SB, scoring balloon.

This study was approved by the ethics committee of our medical institution [Institutional Review Board (IRB) Approval: 260235 and 2026LLWL035]. Given the retrospective nature of the study, the requirement for written informed consent was waived in accordance with institutional regulations. This study was conducted in accordance with the Helsinki Declaration of 1975, as revised in 2024. We have de-identified all patient details. The reporting of this study conforms to the Strengthening the Reporting of Observational Studies in Epidemiology (STROBE) guidelines (18). The completed STROBE checklist is provided as Supplementary Data.

### Endovascular procedure

Both preparation strategies were used in routine practice during the study period, and no uniform device-selection algorithm was applied. The choice of SB or PB was made at the discretion of the treating operator, taking into account patient and lesion characteristics, operator experience, and device availability. The investigators did not influence treatment selection, and patients were classified according to the initial vessel preparation strategy received.

Procedures were performed under local anaesthesia through contralateral femoral arterial access. After placement of a long sheath, angiography was used to assess lesion location, length, stenosis severity, collateral circulation, and distal runoff. Occluded segments were crossed in the direction of blood flow; when antegrade crossing was unsuccessful, a distal runoff vessel was punctured for retrograde recanalisation. Balloon diameter was selected with reference to the adjacent normal vessel. In Group PB, Cordis plain balloons (Cordis, USA) were used sequentially from smaller to larger diameters for lesion predilatation, followed by therapeutic dilation with a Lutonix DCB (Bard, USA). In Group SB, an appropriately sized D-Kutting scoring balloon (DK Medtech, China) was used for lesion preparation, followed by the same Lutonix DCB. Angiographic dissection was assessed after vessel preparation and before DCB inflation. Bailout stenting was performed in cases with severe dissection or residual stenosis > 30% after DCB dilation. Distal angiography before and after treatment was reviewed for distal embolisation and vessel perforation.

All patients received dual antiplatelet therapy with aspirin 100 mg/day and clopidogrel 75 mg/day for at least 3 days before intervention. Dual antiplatelet therapy was continued for at least 6 months after the procedure, followed by long-term single antiplatelet therapy with aspirin 100 mg/day or clopidogrel 75 mg/day.

### Follow-up and study endpoints

Both participating vascular centres used a standardised follow-up protocol. Patients underwent outpatient or telephone follow-up and lower-limb CTA at 1, 3, 6, and 12 months. Intraoperative digital subtraction angiography, follow-up CTA, and demographic data were extracted from the institutional electronic records. All images were independently reviewed by two senior vascular surgeons with 10 years of clinical experience who were blinded to treatment allocation. Disagreements were adjudicated by a third senior vascular surgeon. Medication adherence was assessed during outpatient or telephone follow-up, and imaging and follow-up data were collected using a standardised data-acquisition protocol.

The primary endpoint was any target vessel dissection, comprising mild and severe dissection. Angiographic dissection was classified according to the Kobayashi system: type A, no visible dissection; type B, dissection width < one-third of the lumen diameter; and type C, dissection width ≥ one-third of the lumen diameter (19). Type C was classified as severe dissection and type B as mild dissection. Secondary endpoints were bailout stenting, procedural complications, ankle-brachial index (ABI), 12-month primary patency, and freedom from clinically driven target vessel revascularisation (CD-TVR). Procedural complications were defined as distal embolisation or vessel perforation during the index procedure. ABI was measured on the first day of admission and the day before discharge. Primary patency was defined as the absence of >50% restenosis or occlusion of the target vessel on CTA without repeat intervention. CD-TVR was defined as repeat endovascular revascularisation of the target vessel because of recurrent or worsening clinical symptoms.

### Statistical methods

Python 3.13.5 statistical software were used to analyze the data. To reduce the interference of confounding variables, propensity score matching (PSM) was performed to balance the demographic and lesion characteristics between the two groups. The propensity score for receiving SB was estimated using multivariable logistic regression including age, sex, baseline ABI, coronary artery disease, hypertension, hyperlipidaemia, type 2 diabetes, chronic kidney disease, lesion length, Rutherford category, lesion location, PACSS calcification grade, and number of patent distal runoff vessels. One-to-one matching without replacement was performed within a caliper of 0.20 standard deviations of the logit of the propensity score. Balance-constrained cardinality optimisation was used to retain the largest matched set satisfying an absolute standardised mean difference (SMD) < 0.10 for all modelled baseline covariates. Propensity-score overlap and Love plots were examined graphically. Overlap weighting was applied to the entire 205-patient cohort using the same propensity-score model. Continuous variables were expressed as mean ± standard deviation (SD) or median with interquartile range (Q1-Q3). According to the distribution of the data, independent sample t test or Mann–Whitney U test was used to compare these variables before matching. After matching, these variables were compared using paired t-test or Wilcoxon signed-rank test. Analysis of covariance (ANCOVA) was performed to compare postoperative ABI between the two groups, adjusted for baseline ABI to account for potential baseline imbalance. Categorical variables were expressed as *n* (%), and analyzed utilizing the chi-squared (*χ*2) test or Fisher's exact test before matching. After matching, binomial variables were compared using McNemar's test and multinomial variables were compared using McNemar-bowker's test. Binary outcomes were analysed using conditional logistic regression stratified by matched pair, with odds ratios (OR) and 95% confidence intervals (CI). Relative risks (RR) and absolute risk differences (RD) with 95% CI were additionally estimated using matched-pair clustered generalized estimating equations, and Group SB was used as the reference comparison. Exploratory subgroup analyses of the primary endpoint were conducted for PACSS grade (0–2 vs. 3–4), lesion length (≤150 vs. ＞150 mm), Rutherford category (3 vs. 4–6), and popliteal involvement (absent vs. present) using matched-pair generalized estimating equations with treatment-by-subgroup interaction terms, and Group SB was used as the reference comparison. P for interaction was tested to evaluate whether the treatment effect differed across subgroups. Due to the lack of a sample-size design for interaction, all subgroup analyses were considered to be exploratory and no multiplicity adjustment was applied. The primary patency rate and freedom from CD-TVR rate were calculated using the Kaplan–Meier method, and the comparison between the two groups was performed using the Log-rank test. For time-to-event outcomes, Cox proportional hazards models with robust sandwich variance clustered by matched pair were used to estimate hazard ratios (HR) and 95% CI, and Group SB was used as the reference comparison. Proportional-hazards assumptions were assessed using Schoenfeld residuals. Patients without an endpoint event were administratively censored at the end of the 12-month observation window in the analytic cohort., All tests were two-sided, and *P*-values less than 0.05 were considered statistically significant.

## Results

### Demographic and lesion characteristics

Among 289 potentially eligible patients treated with PB plus DCB or SB plus DCB at the two centres, 44 patients with stenotic rather than occlusive femoropopliteal lesions, 15 patients with bilateral femoropopliteal disease, 11 patients with femoropopliteal in-stent restenosis, 8 patients without complete records, and 6 patients without a patent distal runoff vessel were excluded. No patient was received both PB and SB during the index procedure. The final cohort comprised 205 patients: 126 in Group PB and 79 in Group SB. Baseline demographic and lesion characteristics before matching are summarised in Table 1. Before matching, the largest baseline imbalances were observed for type 2 diabetes, chronic kidney disease, Rutherford category, length of lesion, location of lesion, and PACSS grade when evaluated using SMD. After 1:1 PSM, 63 patients from each group were successfully matched. Post-matching demographic and lesion characteristics are presented in Table 2. All modelled baseline covariates achieved an absolute SMD <0.10 after matching; the maximum absolute post-matching SMD was 0.098 (Supplementary Figure S1). Propensity-score distributions showed common support between treatment groups (Supplementary Figure S2).

**Table 1:** Comparison of baseline characteristics between the two groups before PSM [n (%) ].

Characteristics	PB (*n* = 126)	SB (*n* = 79)	SMD	*P*-value
Age (year, mean ± SD)	77.23 ± 8.76	76.46 ± 8.79	0.088	0.539
Male	96 (76.2%)	59 (74.7%)	0.035	0.807
Comorbidities				
Coronary heart disease	28 (22.2%)	19 (24.1%)	0.043	0.762
Hypertension	33 (26.2%)	24 (30.4%)	0.093	0.515
Hyperlipidemia	20 (15.9%)	14 (17.7%)	0.049	0.729
Type 2 Diabetes	14 (11.1%)	23 (29.1%)	0.461	0.001
Chronic kidney disease	11 (8.7%)	13 (16.5%)	0.234	0.094
Baseline ABI	0.44 ± 0.13	0.44 ± 0.13	0.020	0.125
Rutherford category				0.289
3	39 (31.0%)	16 (20.3%)	0.247	
4	49 (38.9%)	38 (48.1%)	0.187	
5	28 (22.2%)	16 (20.3%)	0.048	
6	10 (7.9%)	9 (11.4%)	0.117	
Length of lesion (cm, mean ± SD)	16.48 ± 4.23	17.34 ± 3.97	0.210	0.148
Location of lesion				0.007
Femoral artery	28 (22.2%)	33 (41.8%)	0.429	
Popliteal artery	62 (49.2%)	25 (31.6%)	0.364	
Femoropopliteal artery	36 (28.6%)	21 (26.6%)	0.045	
PACSS calcification grade				0.018
0	24 (19.0%)	8 (10.1%)	0.255	
1	34 (27.0%)	12 (15.2%)	0.292	
2	28 (22.2%)	28 (35.4%)	0.295	
3	24 (19.0%)	24 (30.4%)	0.265	
4	16 (12.7%)	7 (8.9%)	0.124	
Retrograde recanalisation	28 (22.2%)	18 (22.8%)	0.013	0.925
Distal runoff vessels				0.998
1 distal runoff vessel	92 (73.0%)	58 (73.4%)	0.009	
2 distal runoff vessels	26 (20.6%)	16 (20.3%)	0.009	
3 distal runoff vessels	8 (6.3%)	5 (6.3%)	0.001	

ABI, ankle brachial index; PACSS, peripheral arterial calcium scoring system; PB, plain balloon; PSM, propensity score matching; SB, scoring balloon; SD, standard deviation; SMD, standardized mean difference; n, number of patients; %, percentage.

**Table 2:** Comparison of baseline characteristics between the two groups after PSM [n (%) ].

Characteristics	PB (*n* = 63)	SB (*n* = 63)	SMD	*P*-value
Age (year, mean ± SD)	77.05 ± 9.52	76.21 ± 9.23	0.090	0.709
Male	48 (76.2%)	46 (73.0%)	0.073	0.682
Comorbidities				
Coronary heart disease	14 (22.2%)	16 (25.4%)	0.075	0.676
Hypertension	18 (28.6%)	17 (27.0%)	0.035	0.842
Hyperlipidemia	11 (17.5%)	10 (15.9%)	0.043	0.811
Type 2 Diabetes	12 (19.0%)	10 (15.9%)	0.084	0.639
Chronic kidney disease	5 (7.9%)	5 (7.9%)	0.000	1.000
Baseline ABI	0.44 ± 0.14	0.43 ± 0.13	0.082	0.482
Rutherford category				0.953
3	15 (23.8%)	16 (25.4%)	0.037	
4	29 (46.0%)	27 (42.9%)	0.064	
5	12 (19.0%)	14 (22.2%)	0.079	
6	7 (11.1%)	6 (9.5%)	0.052	
Length of lesion (cm, mean ± SD)	17.17 ± 4.32	16.83 ± 3.90	0.083	0.437
Location of lesion				0.857
Femoral artery	20 (31.7%)	22 (34.9%)	0.067	
Popliteal artery	26 (41.3%)	23 (36.5%)	0.098	
Femoropopliteal artery	17 (27.0%)	18 (28.6%)	0.035	
PACSS calcification grade				0.994
0	7 (11.1%)	7 (11.1%)	0.000	
1	12 (19.0%)	11 (17.5%)	0.041	
2	19 (30.2%)	21 (33.3%)	0.068	
3	18 (28.6%)	18 (28.6%)	0.000	
4	7 (11.1%)	6 (9.5%)	0.052	
Retrograde recanalisation	14 (22.2%)	15 (23.8%)	0.038	0.832
Distal runoff vessels				1.000
1 distal runoff vessel	47 (74.6%)	47 (74.6%)	0.000	
2 distal runoff vessels	12 (19.0%)	12 (19.0%)	0.000	
3 distal runoff vessels	4 (6.3%)	4 (6.3%)	0.000	

ABI, ankle brachial index; PACSS, peripheral arterial calcium scoring system; PB, plain balloon; PSM, propensity score matching; SB, scoring balloon; SD, standard deviation; SMD, standardized mean difference; n, number of patients; %, percentage.

### Primary endpoint

In Group PB, severe dissection occurred in 17 patients (27.0%) and mild dissection occurred in 14 patients (22.2%), yielding an overall target vessel dissection rate of 49.2%. In Group SB, severe dissection occurred in 7 patients (11.1%) and mild dissection in 4 patients (6.3%), for an overall target vessel dissection rate of 17.5% (OR: 5.00, 95% CI: 1.91–13.06; *p* = 0.001) (Table 3). The corresponding RR was 2.82 (95% CI: 1.58–5.04), with the RD of 0.32 (95% CI: 0.17–0.47). In exploratory subgroup analyses, interaction tests were not significant for PACSS grade, lesion length, or Rutherford category. A treatment-by-popliteal-involvement interaction was observed (*p* for interaction=0.018), but given the small number of endpoint events, absence of multiplicity adjustment, and *post hoc* exploratory nature of the analysis, this finding was considered hypothesis-generating only (Figure 3).

**Table 3:** Comparison of study endpoints [n (%) ].

Endpoints	PB (*n* = 63)	SB (*n* = 63)	OR (95% CI)	RR (95% CI)	RD (95% CI)	*P*-value
Target vessel dissection						
Severe dissection	17 (27.0%)	7 (11.1%)	3.00 (1.09–8.25)	2.43 (1.09–5.42)	0.16 (0.03 to 0.29)	0.033
Mild dissection	14 (22.2%)	4 (6.3%)	3.50 (1.15–10.63)	3.50 (1.15–10.63)	0.16 (0.03 to 0.29)	0.027
Total	31 (49.2%)	11 (17.5%)	5.00 (1.91–13.06)	2.82 (1.58–5.04)	0.32 (0.17 to 0.47)	0.001
Bailout stenting	20 (31.7%)	6 (9.5%)	3.80 (1.42–10.17)	3.33 (1.39–8.01)	0.22 (0.08 to 0.36)	0.008
Procedural complications						
Distal embolization	4 (6.3%)	2 (3.2%)	2.00 (0.37–10.92)	2.00 (0.37–10.92)	0.03 (−0.04 to 0.11)	0.424
Vessel perforation	0	0	/	/		
Total	4 (6.3%)	2 (3.2%)	2.00 (0.37–10.92)	2.00 (0.37–10.92)	0.03 (−0.04 to 0.11)	0.424

CI, confidence interval; OR, odds ratio; PB, plain balloon; RD, risk difference; RR, relative risk; SB, scoring balloon; n, number of patients; %, percentage.

**Figure 3:**
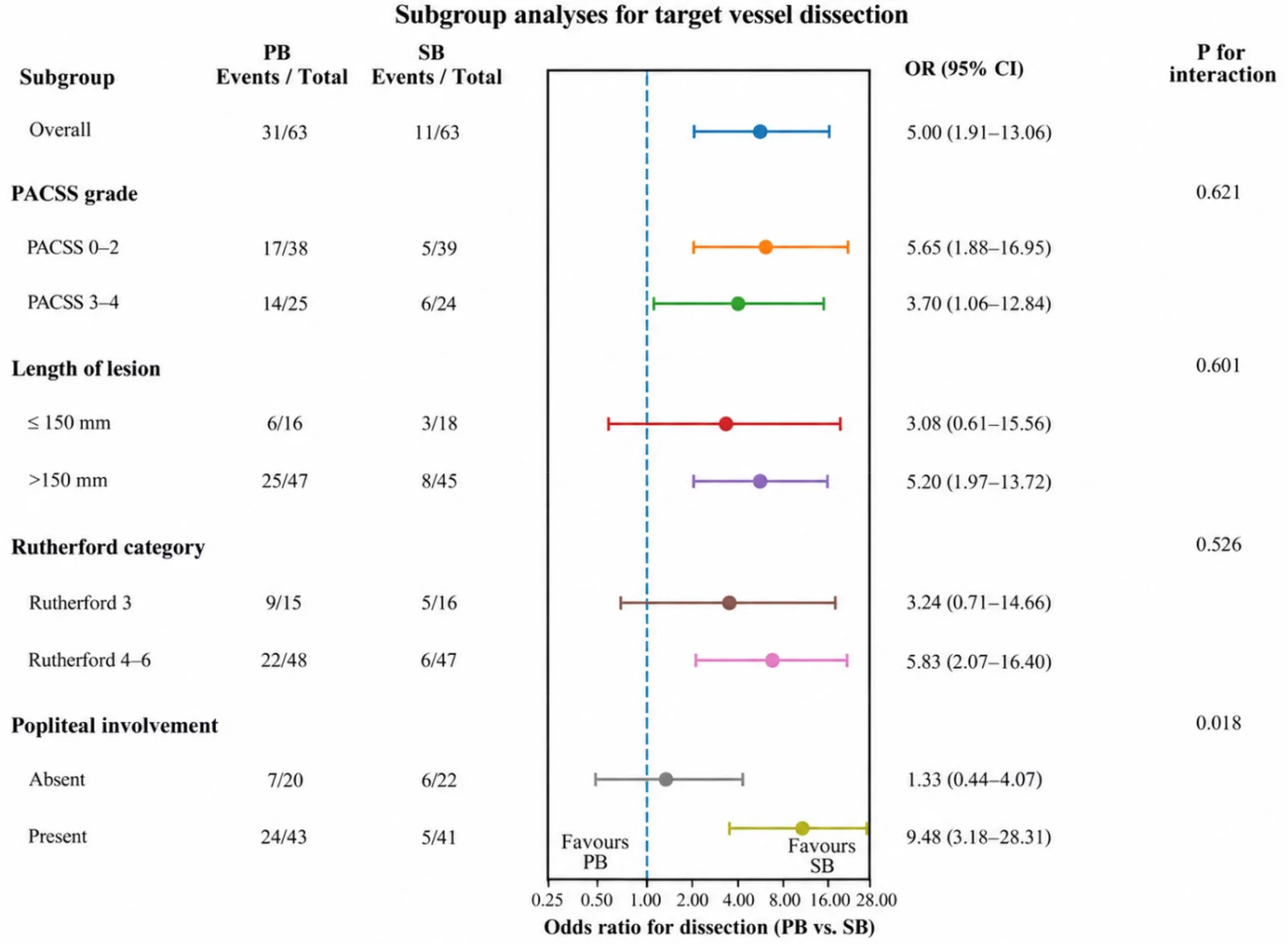
Forest plot of subgroup analyses for the primary endpoint. CI, confidence interval; OR, odds ratio; PACSS, peripheral arterial calcium scoring system; PB, plain balloon; SB, scoring balloon.

### Secondary endpoints

Bailout stenting was required in 31.7% of Group PB and 9.5% of Group SB (OR: 3.80, 95% CI: 1.42–10.17; RR: 3.33, 95% CI: 1.39–8.01; RD: 0.22, 95% CI: 0.08–0.36; *p* = 0.008). Distal embolization occurred in 4 patients in Group PB and 2 patients in Group SB; no vessel perforations occurred. Procedural complication rates were therefore 6.3% and 3.2%, respectively (OR: 2.00, 95% CI: 0.37–10.92; RR: 2.00, 95% CI: 0.37–10.92; RD: 0.03, 95% CI: −0.04–0.11; *p* = 0.008). All distal embolic events were successfully managed by manual aspiration thrombectomy or catheter directed thrombolysis (Table 3).

Postoperative ABI was 1.04 ± 0.15 and 1.02 ± 0.18 respectively, with improvement from baseline in both groups (Table 4). After adjustment for baseline ABI and matched-pair clustering, the adjusted Groups PB-minus-SB mean difference was 0.016 (95% CI: −0.042–0.073; *p* = 0.593). Primary patency at 6 and 12 months were 93.7% (95% CI 84.0%–97.6%) and 87.3% (95% CI 76.2%–93.4%) in Group PB and 96.8% (95% CI 87.9%–99.2%) and 88.9% (95% CI 78.1%–94.5%) in Group SB (HR for loss of primary patency: 1.18, 95% CI: 0.44–3.22; *p* = 0.739) (Figure 4). Freedom from CD-TVR at 12 months were 88.9% (95% CI 78.1%–94.5%) in Group PB and 93.7% (95% CI 84.0%–97.6%) in Group SB (HR for CD-TVR: 1.83, 95% CI: 0.52–6.47; *p* = 0.348) (Figure 5). Schoenfeld-residual tests did not indicate violation of the proportional-hazards assumption for primary patency (*p* = 0.790) or CD-TVR (*p* = 0.861). Full-cohort overlap-weighting sensitivity analyses yielded directionally consistent associations (Supplementary Table S1).

**Table 4:** Comparison of ABI (mean ± SD).

ABI	PB (*n* = 63)	SB (*n* = 63)	Adjusted mean difference	*P*-value
Baseline	0.44 ± 0.14	0.43 ± 0.13	/	0.482
Postoperative	1.04 ± 0.15	1.02 ± 0.18	0.016 (−0.042 to 0.073)	0.593
*P*-value	＜0.001	＜0.001		

ABI, ankle brachial index; PB, plain balloon; SB, scoring balloon; SD, standard deviation.

**Figure 4:**
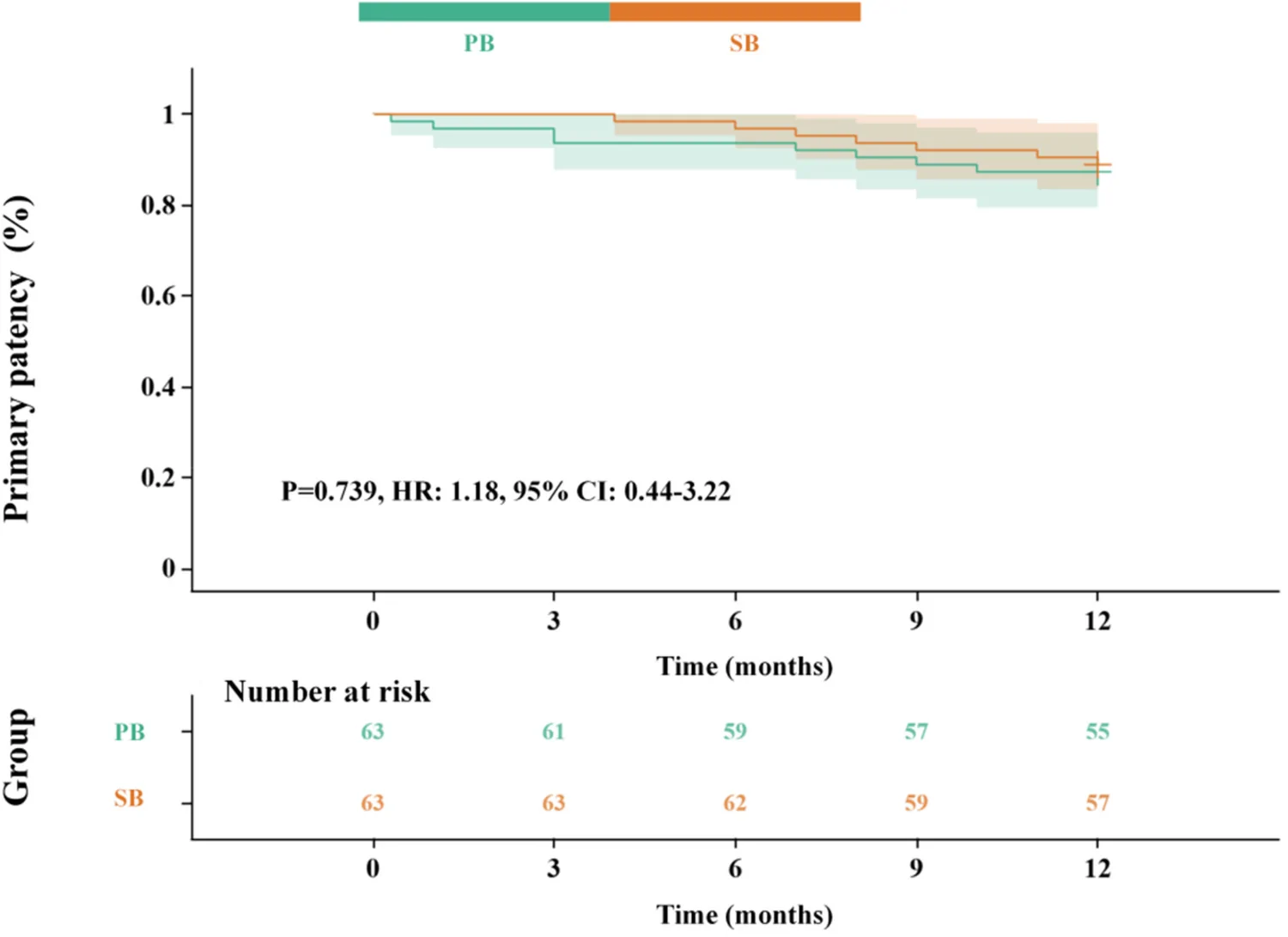
Kaplan–meier analysis of primary patency. CI, confidence interval; HR, hazard ratio; PB, plain balloon; SB, scoring balloon; %, percentage.

**Figure 5:**
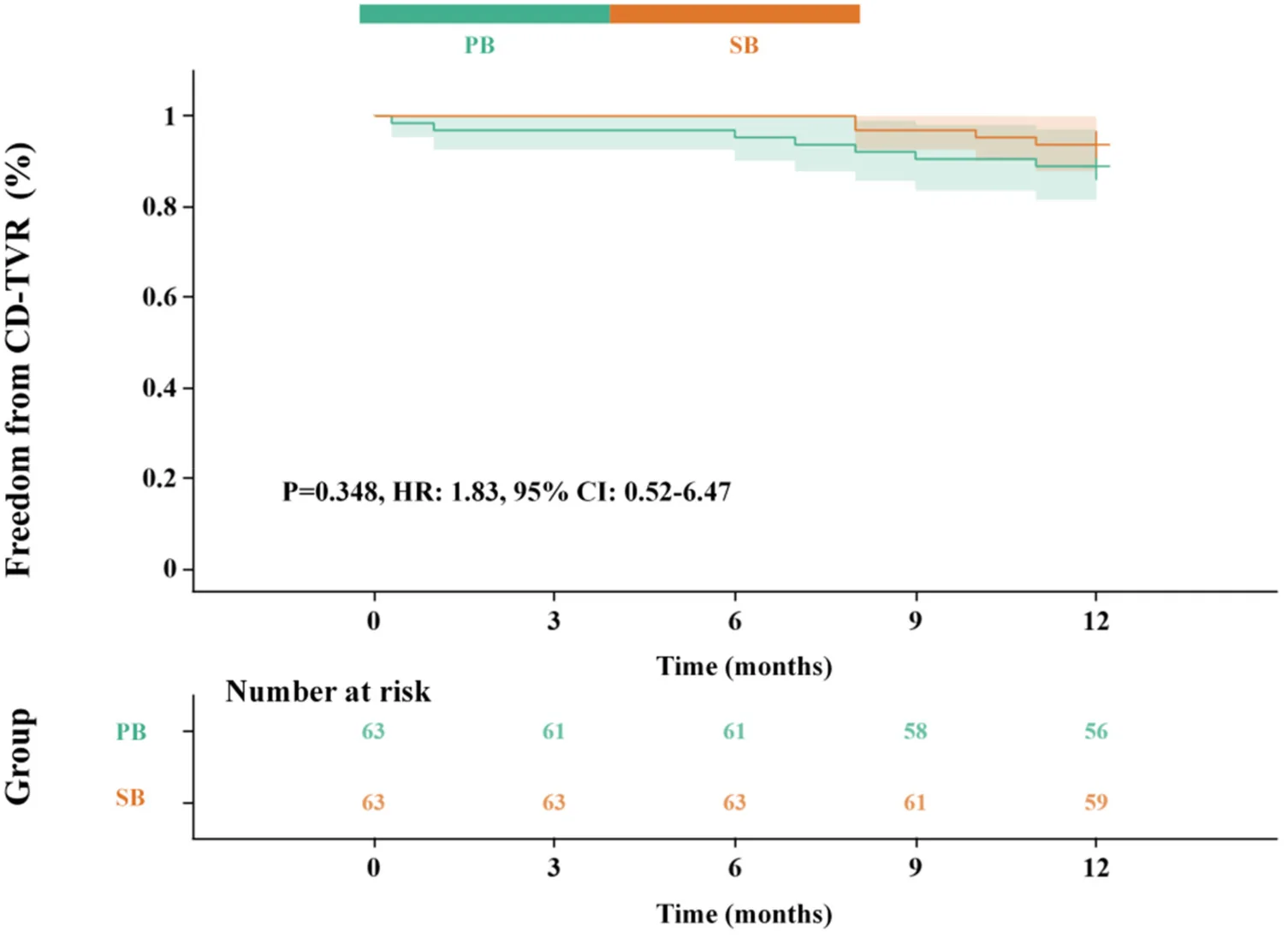
Kaplan–meier analysis of freedom from CD-TVR. CD-TVR, clinically driven target vessel revascularisation; CI, confidence interval; HR, hazard ratio; PB, plain balloon; SB, scoring balloon; %, percentage.

## Discussion

After PSM, SB vessel preparation was associated with a lower rate of target vessel dissection (OR: 5.00, 95% CI: 1.91–13.06; RR: 2.82, 95% CI: 1.58–5.04; RD: 0.32, 95% CI: 0.17–0.47; *p* = 0.001) and bailout stenting (OR: 3.80, 95% CI: 1.42–10.17; RR: 3.33, 95% CI: 1.39–8.01; RD: 0.22, 95% CI: 0.08–0.36; *p* = 0.008) than PB. Its direction was supported by overlap-weighted analysis of the full cohort. Nevertheless, procedural complications, postoperative ABI, 12-month primary patency, and freedom from CD-TVR did not differ significantly between groups.

Post-angioplasty dissection is not merely an angiographic finding; its severity may affect acute lumen gain, the need for bailout stenting, and subsequent patency (20, 21). In 319 femoropopliteal lesions, Kobayashi et al. (19) reported 3-year primary patency rates of 66.0%, 63.8%, and 32.5% in lesions with no, mild, and severe dissection, respectively, and identified severe dissection as independently associated with loss of primary patency. Haraguchi et al. (22) further showed that non-flow-limiting spiral dissection may be associated with lower 1-year patency even when distal antegrade flow is preserved. The value of reducing dissection may therefore extend beyond the prevention of acute flow compromise to the avoidance of extensive or irregular vessel-wall injury. In this study, target vessel dissection and bailout stenting were less frequent after SB than after PB (17.5% vs. 49.2% and 9.5% vs. 31.7%, respectively). Exploratory subgroup analysis and overlap-weighted analysis of the full cohort were also consistent in directionality.

A plausible explanation lies in the mechanical behaviour of the scoring elements. Whereas a PB distributes radial pressure relatively uniformly across the vessel wall, scoring elements concentrate force at discrete contact points, potentially promoting focal plaque deformation or fracture at a lower global balloon pressure while limiting non-target wall stretch and uncontrolled tearing. This interpretation is consistent with reports of favourable procedural performance in calcified coronary lesions (23), arteriovenous fistula and graft stenosis (15), and calcified lower-limb lesions (17), but it remains mechanistic inference because intravascular imaging, quantitative angiography, and pressure measurements were not available in our study. The immediate findings also align with the DASBAD randomised trial (16), in which bailout stenting after DCB preparation was required in 5% of patients treated with AngioSculpt SB and 33% treated with PB. Agreement across a randomised trial and the present real-world cohort strengthens the evidence that SB preparation may reduce acute vessel-wall injury and permanent implant use, although device design, patient selection, and endpoint adjudication differed between studies.

Unlike the immediate procedural endpoints, prespecified procedural complications and postoperative ABI did not differ significantly between groups. Procedural events were uncommon, and the wide 95% CI around the OR for complications (0.37–10.92) indicates limited precision; the findings therefore show no observed increase in distal embolization or vessel perforation with SB but do not establish equivalent safety. ABI improved substantially in both groups, suggesting good overall hemodynamic recovery after DCB treatment, but the reduction in target vessel dissection and bailout stenting requirement by SB did not translate into a higher postoperative ABI. A possible explanation is that the higher rate of bailout stenting in Group PB may have corrected acute flow loss caused by dissection or residual stenosis, thereby narrowing any difference in discharge ABI. Moreover, ABI reflects global limb perfusion rather than local target vessel injury and is influenced by distal runoff, multilevel disease, collateral circulation, and arterial calcification (24). The principal potential advantage of SB in this cohort was therefore improved local vessel preparation and reduced implant use, rather than an additional early increase in ABI after technical success had been achieved.

At 12 months, primary patency and freedom from CD-TVR were numerically higher after SB but did not reach statistical significance. This pattern is consistent with the multicentre real-world analysis by Wang et al. (25), in which specialty-balloon preparation reduced stent implantation without clear differences in periprocedural complications, ABI, or 1-year clinically driven target lesion revascularisation (CD-TLR), and with the study by Kronlage et al. (17), which found no significant difference in 24-month freedom from TLR between AngioSculpt and standard treatment in moderately to severely calcified lower-limb lesions. DASBAD, by contrast, reported higher primary patency with SB, but no significant difference in CD-TLR (16). This apparent divergence is clinically coherent: primary patency captures imaging-detected restenosis even in asymptomatic patients, whereas clinically driven revascularisation requires symptomatic deterioration followed by repeat intervention. DASBAD also included three asymptomatic restenoses in the PB arm and one in the SB arm that contributed to loss of primary patency but not to CD-TLR. In addition, its sample size was based on bailout stenting rather than reintervention, limiting power for the latter endpoint. Several factors may explain why neither primary patency nor freedom from CD-TVR reached statistical significance in the present study. Only 63 matched pairs remained after PSM, and the number of 12-month events was limited, as reflected by the wide CIs despite point estimates favouring SB. The cohort included occlusive lesions across Rutherford categories 3–6 and a broad range of calcification and anatomical complexity, potentially diluting an effect concentrated in selected lesions. More frequent bailout stenting after PB may also have mitigated the consequences of suboptimal vessel preparation, while the use of the same DCB in both groups may have reduced longer-term biological differences. Finally, CTA-based patency assessment in this study differs from the duplex ultrasound definition used in DASBAD. Collectively, available evidence may more consistently favor an immediate reduction in dissection and bailout stenting than a reproducible improvement in mid-term patency or reintervention.

This study is innovative in several ways. First, it provides real-world comparative evidence of specific SB devices and evaluates a single SB platform rather than pooling mechanically heterogeneous specialty balloons. Second, this study uses angiographic dissection, rather than operator-dependent bailout stenting alone, as the primary endpoint. Third, this study combines consecutive two-centre enrolment, PSM, and blinded image review. Fourth, our study examines immediate procedural and 12-month clinical outcomes within the same cohort. The observed divergence between acute vessel preparation and mid-term outcomes is clinically informative and should guide endpoint selection and sample-size planning in future trials.

## Limitations

Several limitations merit consideration. First, treatment allocation was non-random and reflected lesion characteristics, operator experience, and device availability; PSM can balance only measured covariates. Centre, operator, and calendar-period variables were not retained in the analytic dataset and could not be incorporated into the propensity model; residual confounding by indication therefore remains a major limitation. Second, the Kobayashi classification grades dissection by width and does not directly quantify flow impairment; future studies should record dissection morphology, length, lumen compromise, and distal antegrade flow, ideally with independent core-laboratory adjudication, interobserver agreement reporting, and intravascular imaging where feasible. Third, the matched cohort contained only 63 pairs, with few complications and limited 12-month events; consequently, safety, patency, revascularisation, and interaction estimates remain imprecise. Death, major amputation, wound healing, and symptom-improvement variables were not available for competing-risk or broader limb-outcome analyses. CTA-based surveillance also differs from contemporary duplex-ultrasound strategies. Longer, adequately powered prospective studies should focus on long lesions, severe calcification, popliteal involvement, and chronic limb-threatening ischaemia. Finally, the findings relate to the D-Kutting SB and Lutonix DCB, while the safety endpoint was restricted to distal embolisation and perforation; external validation across device platforms should include major adverse limb events, amputation, mortality, wound healing, quality of life, and health-economic outcomes.

## Conclusion

For DCB treatment of complete femoropopliteal occlusions, SB preparation before DCB angioplasty was associated with fewer angiographic dissections and less bailout stenting than PB. No statistically significant difference was observed in the prespecified procedural complications, postoperative ABI, 12-month patency or revascularisation outcomes. The clinical implication is therefore limited to a hypothesis that SB may improve immediate vessel preparation in selected anatomically complex lesions; routine adoption should await prospective evidence based on longer-term clinical endpoints.

## Data Availability

The raw data supporting the conclusions of this article will be made available by the authors, without undue reservation.
