# Supplementary material for: Scoring balloon versus plain balloon vessel preparation before drug-coated balloon angioplasty for femoropopliteal atherosclerotic occlusive disease: a two-centre retrospective cohort study
Source: Front Cardiovasc Med. 2026 Oct 14;13:1975976. doi: 10.3389/fcvm.2026.1975976 (PMC13713448; doi:10.3389/fcvm.2026.1975976)
Supplement: Supplementary file 2 [file Supplementaryfile2.docx]

Supplementary Figure S2. Propensity-score overlap before matching. PB: plain balloon; SB: scoring balloon.
