# Supplementary material for: Scoring balloon versus plain balloon vessel preparation before drug-coated balloon angioplasty for femoropopliteal atherosclerotic occlusive disease: a two-centre retrospective cohort study
Source: Front Cardiovasc Med. 2026 Oct 14;13:1975976. doi: 10.3389/fcvm.2026.1975976 (PMC13713448; doi:10.3389/fcvm.2026.1975976)
Supplement: Supplementary file 3 [file Table1.docx]

Supplementary Table S1. Full-cohort overlap-weighting sensitivity analysis.

| Endpoints | Weighted PB risk | Weighted SB risk | RR  (95% CI) | RD  (95% CI) | *P*-value |
| --- | --- | --- | --- | --- | --- |
| Target vessel dissection | 40.6% | 20.7% | 1.96 (1.17-3.28) | 0.20 (0.06 to 0.33) | 0.011 |
| Bailout stenting | 26.7% | 11.8% | 2.27 (1.09-4.70) | 0.15 (0.03 to 0.27) | 0.028 |
| Procedural complications | 4.3% | 3.3% | 1.30 (0.28-5.91) | 0.01 (-0.05 to 0.07) | 0.738 |

CI: confidence interval; PB: plain balloon; RD: risk difference; RR: relative risk; SB: scoring balloon; %: percentage.
